# Fish Species Composition, Distribution and Community Structure in Relation to Environmental Variation in a Semi-Arid Mountainous River Basin, Iran

**DOI:** 10.3390/w14142226

**Published:** 2022-07-14

**Authors:** Mojgan Zare-Shahraki, Eisa Ebrahimi-Dorche, Andreas Bruder, Joseph Flotemersch, Karen Blocksom, Doru Bănăduc

**Affiliations:** 1 Department of Natural Resources, Isfahan University of Technology, Isfahan 84156-83111, Iran; 2 Institute of Microbiology, University of Applied Sciences and Arts of Southern Switzerland, 6850 Mendrisio, Switzerland; 3 Office of Research and Development, United States Environmental Protection Agency, Washington, DC 20460, USA; 4 National Health and Environmental Effects Research, United States Environmental Protection Agency, Corvallis, OR 97333, USA; 5 Applied Ecology Research Center, Lucian Blaga University of Sibiu, European Union, 550012 Sibiu, Romania

**Keywords:** natural habitat variability, human impact, fish, Karun basin, conservation, ecosystem management

## Abstract

We analyzed spatial variation in fish species richness and community composition in the Karun River basin, Iran. Knowledge about fish diversity in the basin is incomplete and varies widely along spatial and temporal scales: The Karun is the longest river in Iran (950 km) with the largest drainage area (about 67,000 km^2^). Fish samples were collected from 54 sites from July through August 2019 using a backpack electro-fisher. Physico-chemical and habitat parameter data collected at each site included pH, conductivity (μS/cm), dissolved oxygen (mg/L), water temperature (°C), turbidity (NTU), stream width (m), stream depth (m), water velocity (m/s) and elevation (m). In total, 37 species were collected (5241 individuals weighing 110.67 kg). The species collected represented 12 families and 27 genera. A total of 13 endemic species (35.14%), 16 native species (43.24%), and eight non-native species (21.62%) were recorded. Diversity indices were calculated and used to measure the spatial variation in community composition. Relationships between native and endemic species assemblage structure and environmental descriptors were assessed using canonical correspondence analysis (CCA). The first two axes of the canonical correspondence analysis explained 62.57% of the variation in the data. Of the nine environmental descriptors analyzed, eight significantly affected species distribution; however, electrical conductivity and elevation were most influential. Our study provides up-to-date status information on the distribution of freshwater fishes in the Karun River basin. This information is essential for developing conservation and management strategies to support the long-term sustainability of fish populations in the Karun River basin.

## 1. Introduction

Large river basins have been inhabited by humans for more than five millennia and have contributed to the success of some of the most important human culture and civilization centers in human history [1–5]. However, human presence in the watersheds resulted in complex and highly variable impacts on lotic ecosystems. Such human impacts have altered water flows and the quality of habitats of many freshwater fish species and are a major cause of the decline in freshwater fish biodiversity [3–5]. Despite the importance of these impacts, many large river systems have not been adequately studied, especially where the spatial and temporal dynamics of the area require complex study designs for robust assessment [6]. Many Iranian rivers, such as the Karun River basin, serve as examples of this lack of knowledge [7–10].

The Middle East is a transition region between three important biogeographical units, the Palearctic, the Afrotropical, and the Oriental realms [11]. Iran is located in the Palearctic region bordering the Oriental and Ethiopian zones [12], and its north-west, west and south-west are parts of Irano-Anatolian biodiversity hot spot with high biodiversity and endemism, especially with regard to freshwater fish [13,14]. The ichthyofaunal composition of Iran is a result of the Iranian Plateau boarding the Eastern Mediterranean (Western-Palearctic), the Southern Asian (Indo-Oriental) and the Ethiopian regions [15]. As a result, this area is considered as the origin of many fish species, and an important crossroad of migration routes, resulting in high biodiversity of freshwater fishes [16,17]. New species of fish are regularly being described from this area. However, human population growth, aquaculture, fish introductions and movement, drought, pollution and habitat destruction have had negative effects on the diversity of freshwater fish communities [15,18].

Among the Iranian Plateau drainage systems, the Karun River basin shows a great fish diversity [13,14,19] despite being affected by pollution and impacted water quality [20], which has increased the environmental risks to freshwater fish [21]. For example, many sections of the basin receive raw sewage from industrial, agricultural and urban sources which may lead to the bioaccumulation of chemicals in fish tissues [22]. Negative effects of water quality issues or loss of natural habitats on aquatic organisms may include effects on reproduction, behavior, the immune system or genetic damage leading to alterations in community composition [23]. Understanding the impact of such pollutants on fish species composition, distribution and community structure in the Karun River basin is challenging due to limited knowledge on fish diversity and distribution in the different sections of the basin. Thus, the goal of our study was to reveal spatial patterns of fish community structure in the Karun River basin in the context of environmental variables.

## 2. Material and Methods

### 2.1. Study Area

**Figure 1.**
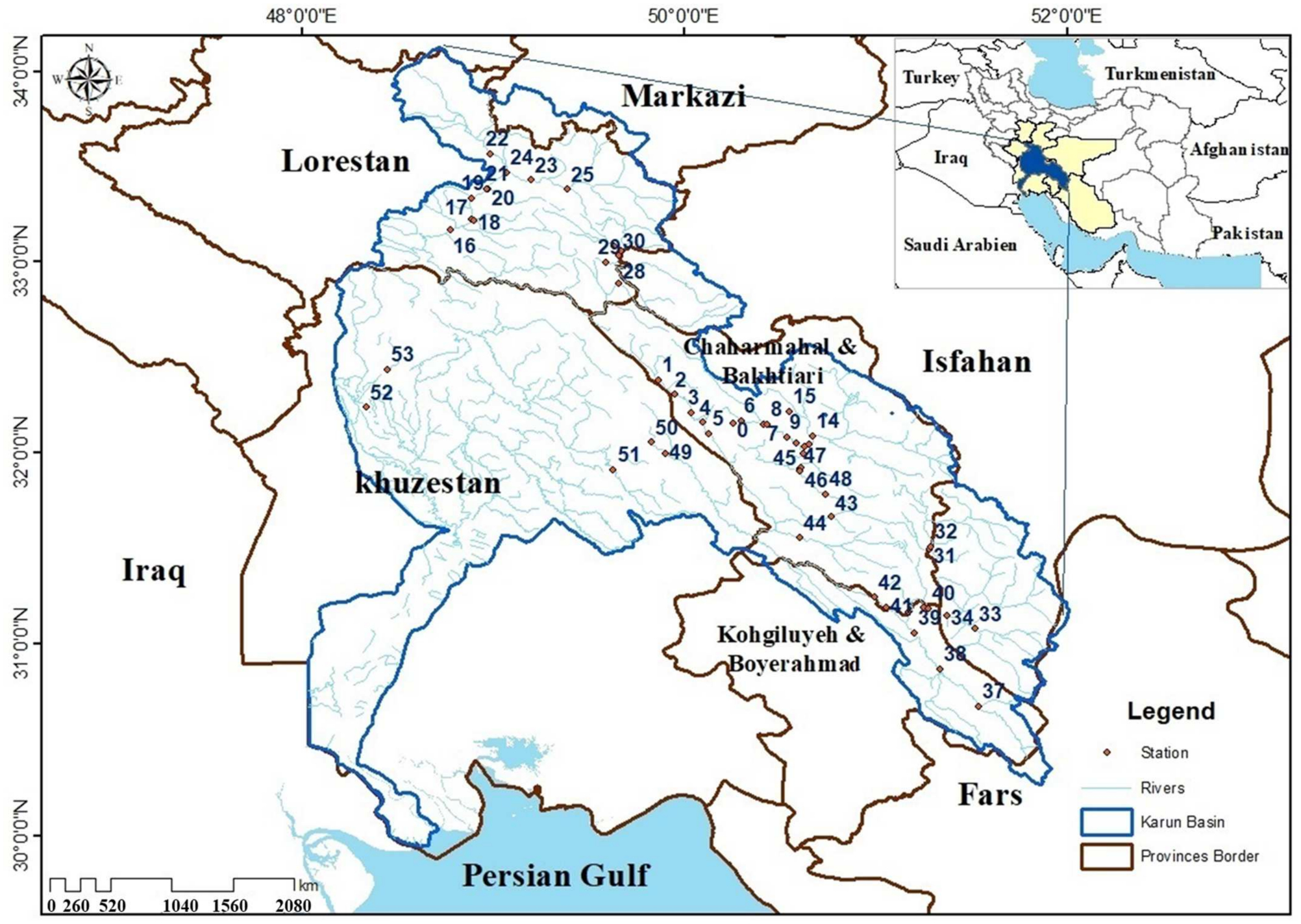
Distribution of sampling sites (0–53) in the Karun River basin, Iran.

The Karun River is the longest river (950 km) in Iran with the largest drainage area (about 67,000 km^2^). It flows from the central Zagros range and discharges into the Persian Gulf. This study was limited to wadable sections of Karun’s basin, including 18 large and small rivers (Figure 1). The average distance between sampling sites was 27 ± 44 km. Where present, the tree vegetation on both sides of the stream was mixed, mostly consisting of Fagaceae, Tamaricaceae, and Salicaceae.

### 2.2. Field Sampling

Fish samples were collected from 54 sites (Figure 1) from July to August 2019 using a backpack electro-fisher (Samus 1000, Poland; 12 V import, 250 V export), which was applied from downstream to upstream at each site. Sampling sites were 150–200 m long and comprised different mesohabitats. Each site was fished for approximately 90 min. Fish with body lengths greater than 20 mm were identified to the species level, counted, measured for total length and weight, and returned to the river [24]. Fish less than 20 mm were conserved in formaldehyde and transported to the laboratory for identification using a dissecting microscope. The identification of fish species was based on available references [12,14,25,26].

### 2.3. Physico-Chemical and Habitat Parameters

Physico-chemical and habitat parameters measured in situ included pH, electrical conductivity (EC) (μS/cm), dissolved oxygen (DO: mg/L), water temperature (T: °C), turbidity (NTU), stream width (m), average stream depth (m), water velocity (m/s) and elevation (m). Dissolved oxygen was measured by a portable oxygen meter (Model: WTW oxi 3210); stream width and stream depth using measurement tapes and tube, respectively; water velocity (Flow meter, Model: 001); and elevation using GPS (Garmin GPSMAP 64X).

### 2.4. Data Analysis

Dominant and common fish species were determined by the index of relative importance (*IRI*) based on the numerical percentage, weight percentage and frequency of occurrence Equation (1) [27]:

(1) $$IRI_{i}=\left(\%N_{i}+\%W_{i}\right)\times \%F_{i}$$

where %*N*_*i*_ and %*W*_*i*_ represent the percentage in terms of numbers and percentage in terms of weight, respectively, of species *i* in the total catch, and %*F*_*i*_ is the frequency of occurrence of species *i*. When *IRI*_*i*_ was greater than 10%, species *i* was considered dominant, whereas species with 1% < *IRI*_*i*_ < 10% were considered common.

Several diversity indices Equations (2)–(5) were used to measure the spatial variation in fish species diversity as follows [28,29]:

(2) Margalef species richness index : D=(S−1)/lnN

(3) $$\text{Simpson}’\text{s index of diversity}:D=1-\sum {\left(Pi\right)}^{2}$$

(4) $$\text{Shannon–Wiener diversity index }:\;H^{\prime }=-\sum Pi\;ln\;Pi$$

(5) $$\text{Pielou evenness index }:\;J^{\prime }=H^{\prime }/lnS$$

where *S* is the number of species, *N* is the total number of individuals of all species, and *Pi* is the proportion of each species in the sample.

A dataset covering all collected species at each site was constructed. Similarity analyses were conducted based on the relative abundance of species/site. The furthest-neighbor method with squared Euclidean distance was then used for cluster analysis of the community matrix. A gradient in the community of native and endemic species and the importance of environmental descriptors were assessed using canonical correspondence analysis (CCA). Species with a frequency of occurrence of at least 10% of the total sampled sites were included in this analysis (9 native and 11 endemic species). Statistical analysis was carried out using R software (version 4.0.3) [30] in the *vegan* package.

## 3. Results

### 3.1. Species Composition

Thirty-seven species in total were collected from the 54 sites (5241 individuals weighing 110.67 kg) and categorized into 12 families and 27 genera (Appendices A and B). Of these, the most species-rich family was Cyprinidae (40.5%, 15 species), followed by Leuciscidae (21.6%, eight species), Nemacheilidae (10.8%, four species) and Xenocyprididae (5.4%, two species). Aphanidae, Poeciliidae, Sisoridae, Mastacembelidae, Salmonidae, Mugilidae, Gobionidae and Gobiidae were represented by one species each. A total of 13 endemic species (35.14%), 16 native species (43.24%) and eight non-native species (21.62%) were reported in the Karun River basin. The fish from the study area belonged to four feeding groups. The percentage of omnivorous, benthivorous, carnivorous, and herbivorous fish accounted for 54.05%, 21.62%, 8.11% and 16.22%, respectively. The substrate preference for most of the species was rocky streambed (72.97%), followed by vegetative substrate (27.03%). Distribution and presence status of different species at the Karun River basin, along with other characteristics, are presented in Table 1. The dominant species were *Capoeta coadi* (IRI, 23%), followed by *Capoeta aculeata* (IRI, 12.41%), *Garra rufa* (IRI, 10.29%) and *Chondrostoma regium* (IRI, 10.27%). The common species were *Alburnus sellal* (IRI, 6.78%), *Capoeta pyragyi* (IRI, 5.72%), *Squalius berak* (IRI, 2.77%), *Capoeta trutta* (IRI, 2.54%), *Garra gymnothorax* (IRI, 2.25%), *Alburnoides idignensis* (IRI, 1.22%), and *Barbus lacerta* (IRI, 1.02%) (Table 2). The abundance and biomass of these 10 species accounted for 78.57% of the total individuals and 83.86% of the total biomass.

### 3.2. Species Distribution in the Karun River Basin

**Figure 2.**
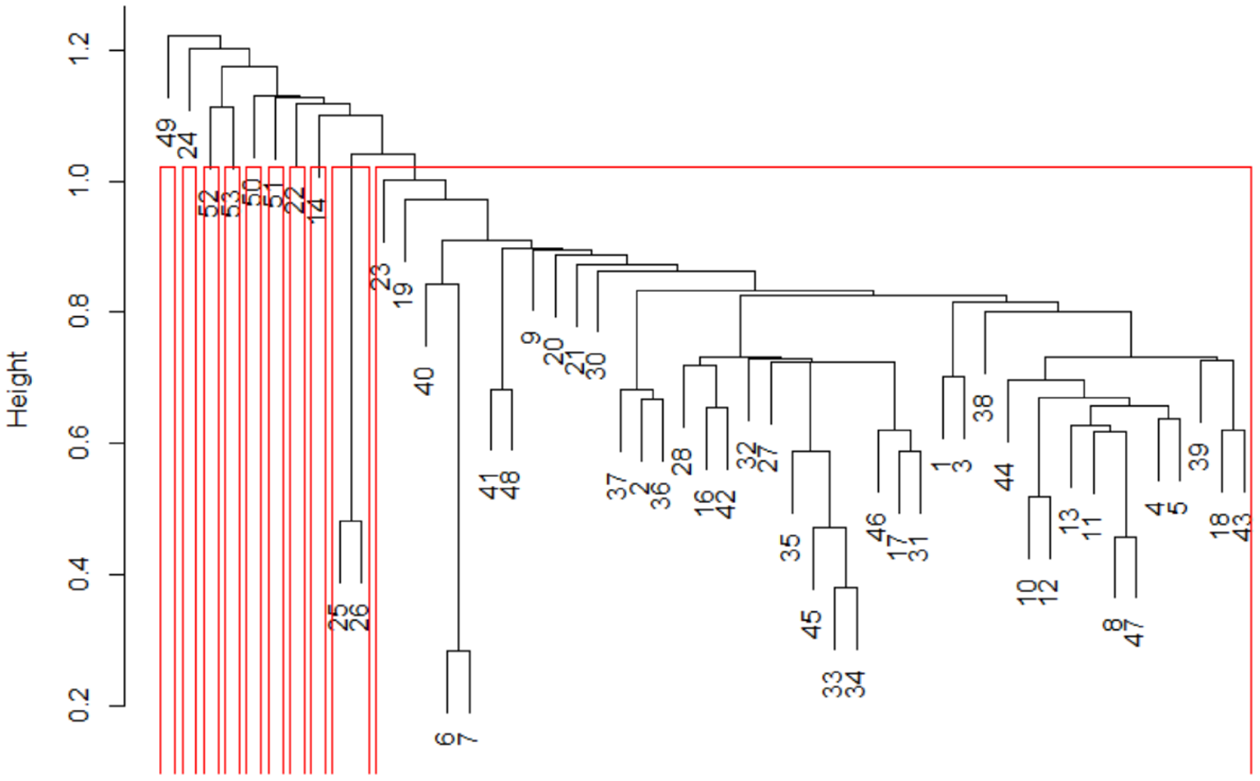
Grouping data matrix rows (sampling sites) using cluster analysis and silhouette width.

**Figure 3.**
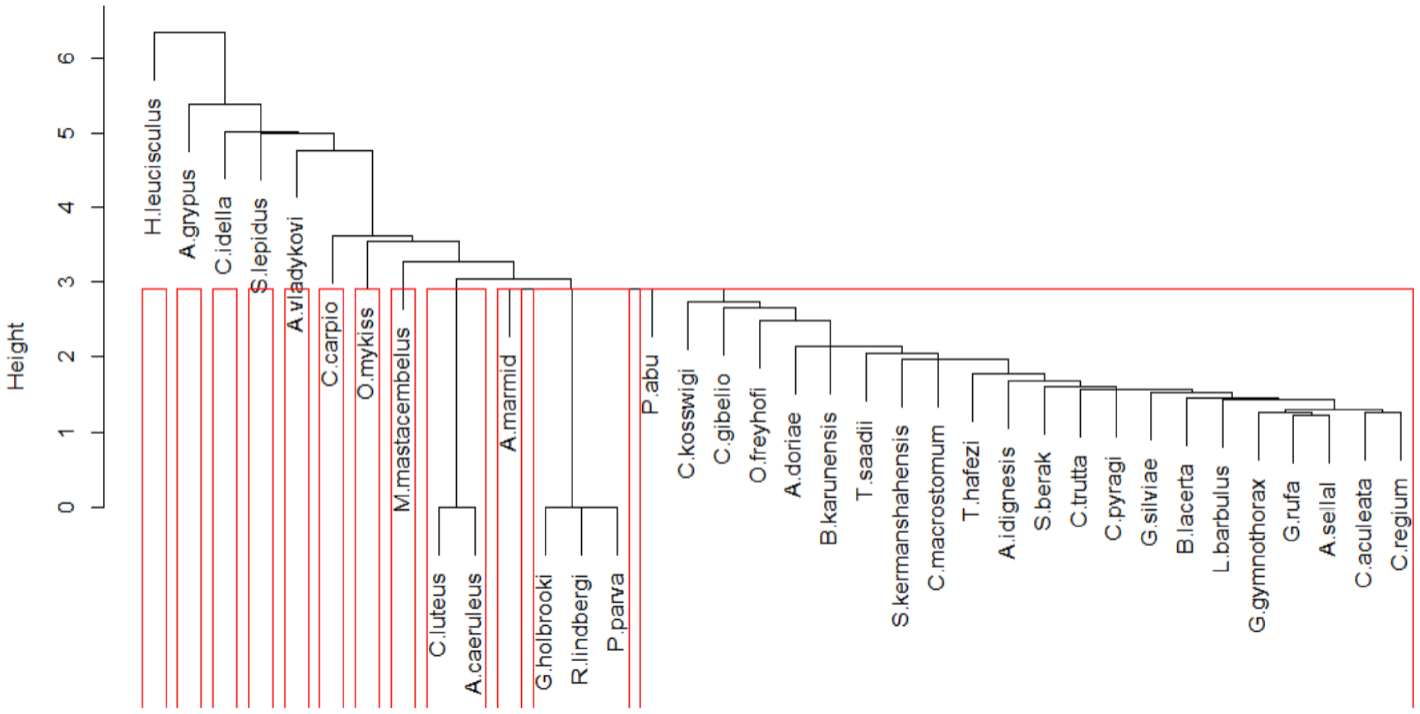
Grouping data matrix columns (fish species) using cluster analysis and silhouette width.

The general distribution of fish species in the 54 sites is shown in Appendix B. Four species (*Capoeta coadi*, *Chondrostoma regium*, *Garra rufa*, and *Alburnus sellal*) appeared in more than 50% of sites, whereas nine species were recorded in only one or two sites. Cluster analysis divided sampling sites into ten different groups (Figure 2) based on relative abundance of different fish species. Most sampling sites, and consequently, most fish species were in one group, all of which were located in the upper and middle parts of the Karun River basin (Figure 3). The other groups covered five sites (49–53) located in the lower mainstream regions in the Karun River basin (Figure 2). Five species (*Mastacembelus mastacembelus*, *Carasobarbus luteus*, *Arabibarbus grypus*, *Alburnus caeruleus* and *Hemiculter leucisculus*) were only reported from these sites. Some species such as *Gambusia holbrooki*, *Rhinogobius lindbergi* and *Pseudorasbora parva* were found only at site 22 and *Ctenopharyngodon idella* was present only at site 24.

### 3.3. Spatial Variation in Fish Composition

Fish diversity and evenness indices are presented in Table 3. No fish were caught at sites 0, 15, and 29, which are therefore not included in Table 3. The highest species richness was observed at sites 20 and 22 (with 13 and 15 fish species, respectively), whereas the lowest value (one species) was observed at site 49. The maximum abundance (388 individuals) was collected at site 38, whereas the minimum (two and three individuals) was observed at sites 18 and 49, respectively. The highest biomass (5688.4 g) was observed at site 10, whereas lowest (14.08 g) was observed at site 49. The species diversity indices also differed among sampling sites. The Simpson dominance index ranged from 0–0.86, with a smaller value indicating a higher concentration and lower diversity. The maximum value for Margalef species richness index (2.96), Shannon−Wiener diversity index (2.14), Simpson’s index (0.86), and Pielou evenness index (1) were observed at sites 31, 22, 41 and (18, 46) respectively. Site 49, with only one species, had the minimum score (0) for evenness and diversity indices.

### 3.4. Environmental Variables

Details of some measured physico-chemical and habitat parameters in the Karun River are presented in Table 4.

**Figure 4.**
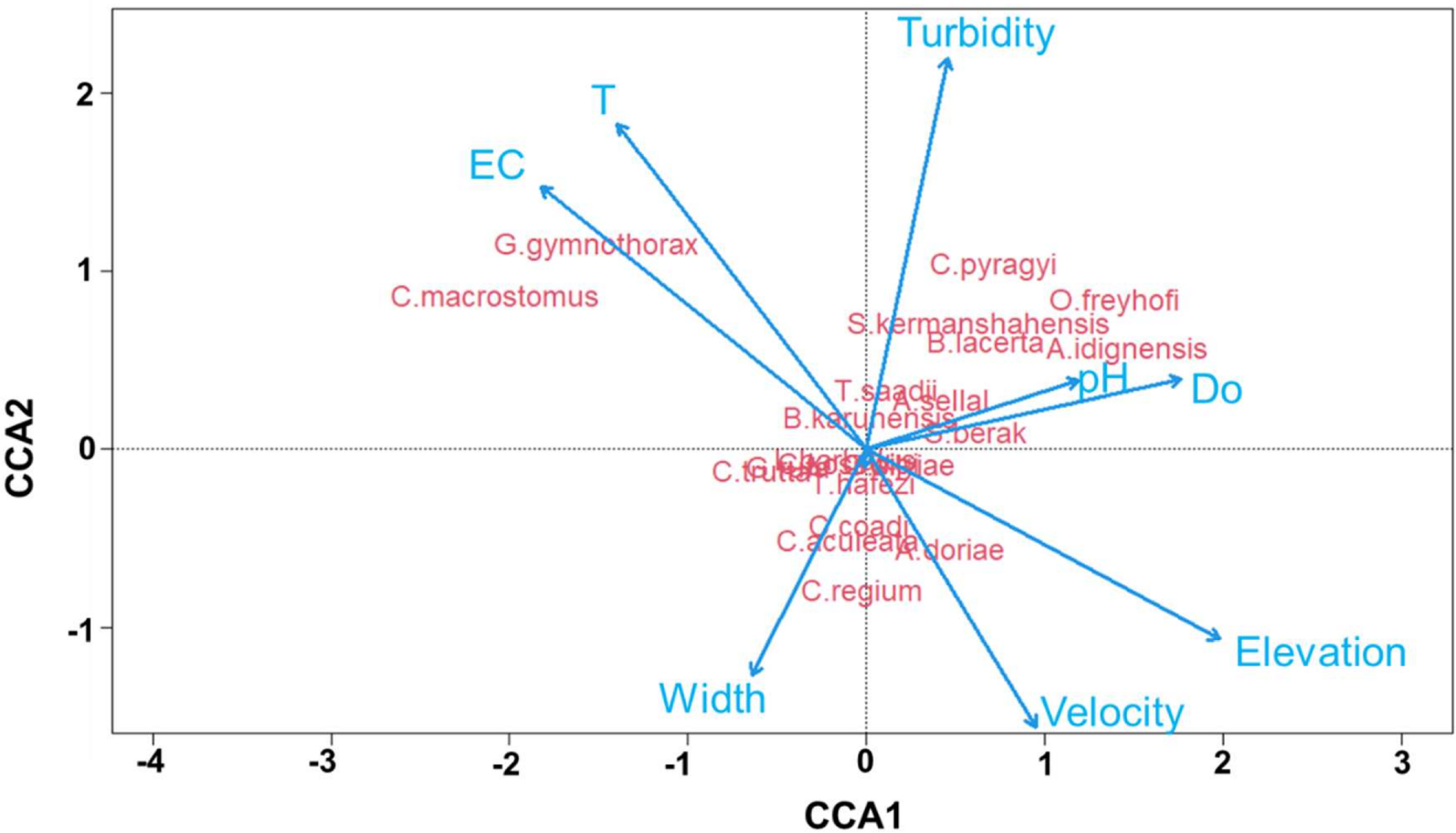
CCA biplot for native and endemic fish species composition and environmental variables. (Abbreviation, EC: Electrical conductivity, T: Temperature, Do: Dissolved oxygen).

The first two axes of the canonical correspondence analysis explained 62.57% of the data variation. The first axis explained 37.17%, and the second axis explained 25.4%. Out of nine analyzed environmental descriptors, eight variables had a significant influence on species distribution (Table 5), but electrical conductivity and elevation were the most influential. In streams with greater stream width, *C. trutta*, *C. coadi*, *C. aculeata*, *C. regium* and *G. rufa* were more common and some species, such as *G. silviae*, *A. sellal*, *T. hafezi*, *C. kosswigi*, *L. barbulus* and *T. saadii*, were present in shallower depths. In rivers with higher electrical conductivity and temperature, the most common species were *C. macrostomus* and *G. gymnothorax*; in rivers with higher water velocity and elevation, *A. doriae* and *S. berak* were common (Figure 4).

## 4. Discussion

### 4.1. Environmental Parameters

The influence of environmental variables on fish species distribution and community structure contributes to a more complete understanding of fish-habitat relationships [31]. Among water quality parameters, water temperature (T) is one of the most important parameters that affects the survival, growth, and metabolic activities of fish [31–34]. The maximum recommended level of water temperature in some references is 20 °C to 30 °C to support fish growth rate [35–38], which was consistent with our results. In our study, water temperature increased longitudinally from headwaters to downstream sites [36–38]. pH was probably also influential in explaining the presence or absence of fish species. The optimal pH for freshwater fish species usually ranges from 5.5 to 7.5 [31–34], which is consistent with the results of our study. The concentration of dissolved oxygen also strongly influences abundance, distribution, activity, behavior and survival of freshwater fish [39–41]. In this study, high concentrations were consistently recorded (Table 4). Water turbidity and velocity can also impact fish community structure. Water transparency in flu-vial systems is affected by season, rainfall patterns, and water velocity [42], whereas current velocity is controlled by season, altitude, and morphological structure [42]. Meteorology and microclimatic drivers also influence hydrology of the study sites in the Karun with depth of water increasing from upstream to downstream sites. High turbidity and high flow velocities were indeed observed in the Karun, in particular during and after rainfall events. Turbidity values reported as best supporting fish communities range from 0–40 (NTU) [43]. In our study, increased water velocities were associated with decreases in all diversity indices and in richness, a finding concurrent with those of other studies [36,44–46].

### 4.2. Fish Community Structure and Diversity

Our study provides information about the community structure and spatial variation of the fish species in the Karun River basin. Fish species vary in their sensitivity to human intervention, natural calamities and environmental degradation in general [42,47–50]. The majority of fish species observed in our study belonged to the Cyprinidae and Leuciscidae families. The most dominant species was *C. coadi*, followed by *C. aculeata*, *G. rufa* and *C. regium*. These species have many populations across their distribution range and no known major threat; therefore, they were classified as species of least concern [14]. Owing to the large size of two of the species in the genus *Capoeta*, they are targeted by fishermen. *C. regium* is an endangered species in Turkey [51].

Endemic freshwater fish comprise 79 species in Iran [14], of which thirteen species are endemic to the Karun basin (Table 1). Endemic species have, by definition, a small geographic spread and often depend on specific and sometimes rare habitat types [52,53]. Among them, some species, including *Aphanius vladykovi* and *Sasanidus kermanshahensis*, were classified as near threatened and endangered, respectively [14]. The endemic species we detected had limited distribution in our set of sites (Appendix A) and are particularly sensitive to change and degradation of the environment compared to more dispersed species [51,52]. Only *Carasobarbus kosswigi* (native) and *Cyprinus carpio* (non-native) were considered vulnerable [14].

**Figure 5.**
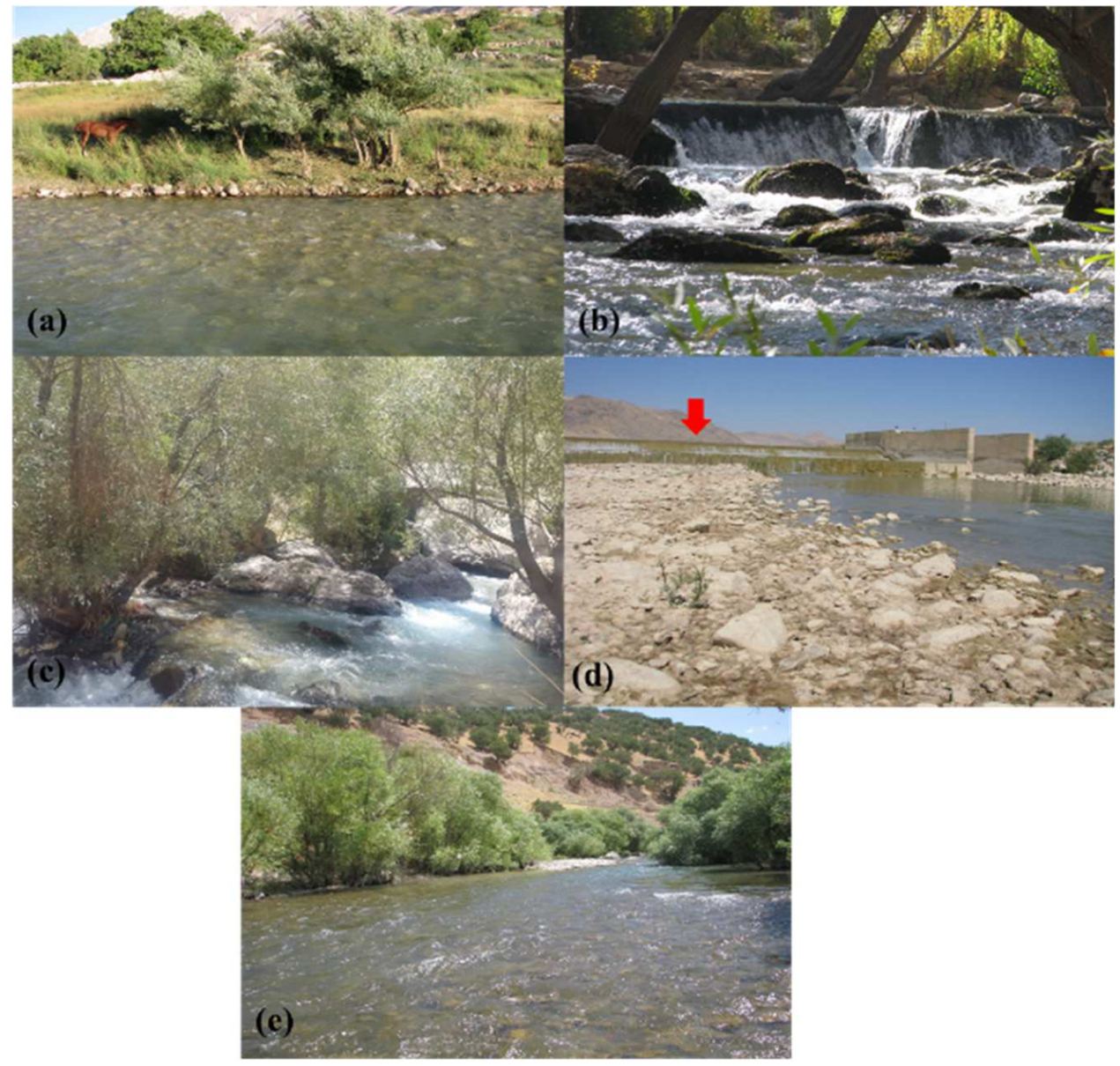
Some examples of sampling sites in the Karun River basin; (**a**) site 0 (Dezdaran), (**b**) 15 (Cheshmeh Pireh ghar), (**c**) 29 (Ab sefid waterfall), (**d**) 22 (Tireh), (**e**) 20 (Chamchit 1).

Site 22 had the highest species richness among all sampling sites. This was surprising given that site did not have water quality and habitat conditions that seemed appropriate to support the observed fish community. We hypothesize that the observed high richness may have been partly caused by a great flood event that occurred in the study area in the early spring of 2019 prior to our summer sampling, and that some species may have been transferred to this site and were not able to return to their original habitat when waters receded due to the presence of a previously submerged barrier in the river (Figure 5d). Additionally, three non-native species (i.e., *Pseudorasbora parva*, *Rhinogobius lindbergi* and *Gambusia holbrooki* were recorded only at this site. Site 20 (Figure 5e) supported the next highest species richness. Bank areas were well vegetated and a mixture of micro-habitats was present. Physico-chemical characteristics and habitat conditions were also suitable. The lowest species richness was observed at site 49 with only one species, *Hemiculter leucisculus*. This species is typical of downstream sites of the Karun River basin and generally not observed in upstream areas [14]. Hydrological characteristics of site 49 varied substantially over the day due to the influence of water discharge from power generation turbines. When discharges occur from the reservoir into the river during power generation, the mean depth, water velocity and flow increase substantially. Regularly disrupted flow conditions probably limited fish diversity at this site. Nyanti et al. (2018) reach a similar conclusion in the context of hydropower operations on the Batang Ai river in Malaysia. Evenness in our communities was close to 1, indicating very few dominant species in the Karun River basin (Table 3) [53]. Among the sampling sites, sites 0 (Figure 5a), 15 (Figure 5b) and 29 (Figure 5c) were registered as fish-free sites. Observed conditions that might have contributed to these sites being less utilized by fish include low water temperature, high water velocity, the presence of large boulders in the riverbed, and also the lack of nutrients. This, together with reduced connectivity of these sites, might explain the absence of fish at these three sites [54,55].

In general, the results of cluster analysis (Figures 2 and 3) showed that some species, such as *Hemiculter leucisculus*, *Carasobarbus luteus*, *Mastacembelus mastacembelus*, *Alburnus caeruleus* and *Acantobramam marmid*, were found only in downstream parts of the Karun River basin (Sites 49–53). These species are generally considered tolerant species that prefer warm water with stony or gravel substrates and bushy riparian zones [56]. Environmental conditions differ strongly between the lower and the upper parts of the Karun River basin (e.g., water depth, river width, substrate size, temperature, EC, pH, etc.). The lower parts of the basin are furthermore influenced by urbanization. These factors influence the distribution of fish in rivers [36].

In this study, the CCA analysis revealed how native and endemic fish community composition responded to changes in environmental variables in the Karun River Basin [57,58]. Most of the measured environmental variables had a significant influence on species distribution (Table 5). However, EC and elevation were the most influential variables for the distributions of native and endemic fish species in the Karun River basin. *G.gymnothorax* and *C.macrostomus* were positively associated with high conductivity, whereas *S.berak*, *A.sellal*, *T.saadii* and *B.karunensis* were positively associated with dissolved oxygen concentrations. Jaramillo-Villa et al. (2010) and Suarez et al. (2011) stated that altitudinal gradients promote changes in community composition along river systems due to differences in habitat use, feeding behaviour and movement of fish species [59,60]. Likewise, Dubey et al. (2012) observed that EC, DO, pH, alkalinity, and salinity were most strongly correlated with fish community composition of the Kali Gandaki River basin in Nepal, and the Ganga River basin in India [61]. In the study of Mondal and Bhat (2020) EC, DO and water velocity were influential factors in tropical streams in India [62]. Our results concur with the findings from these studies and further support the importance of these environmental variables in characterizing fish–environment relationships.

### 4.3. Current Threats to Fish Communities in the Karun River Basin

Disturbances due to drought, dam construction, sand excavation (i.e., damaging effects on fish feeding, migration, and reproduction grounds), pollution, and overfishing are the most significant threats to fish biodiversity in Iran [25,63,64] as in other parts of Asia [65–67]. For example, the construction of the Karun 1, 3, 4, Abbaspour, and Gotvand dams on the Karun River has strongly altered river connectivity and hydrology, and disrupted the longitudinal migration of fishes. In particular, the frequent droughts in the last few years have severely threatened aquatic organisms including fish. In summer, many large rivers are reduced to a trickle as a result of excessive water abstraction for agricultural purposes. However, it seems that some fish species have been able to adapt to these new conditions and persist. Overfishing and illegal fishing are other threats to fish communities throughout all large river systems in Iran, especially in the downstream parts of the Karun River basin. As a result of such widespread alterations and habitat loss, fish communities have been negatively impacted in most Iranian water bodies [25].

## 5. Conclusions

Conservation of freshwater fish should be based on a comprehensive understanding of large-scale species-richness patterns and endemism patterns. The methods used in our study provide a basis for assessing the current status of freshwater fish diversity in the Karun River basin. This status information is essential in determining appropriate conservation and management strategies and filling gaps in knowledge in important but strongly altered basins such as the Karun River basin. Some of the described impacts of altered environmental conditions with consequences on fish community composition could be alleviated by the designation and effective management of protected areas. Based on our findings, we propose the following conservation measures to protect and sustainably use fish biodiversity in the Karun River basin: (1) re-establishment of economically important fish species such as *L. barbulus*, *A. grypus and C. kosswigi*; (2) prohibition of fishing during the breeding season; and (3) habitat restoration for endangered and important species such as *G. silviae* and *S. kermanshahensis*.

## Figures and Tables

**Table 1.** Different characteristics of fish species recorded in the Karun River basin.

Species	Distribution	Presence Status in Karun Basin	Feeding Behaviour	Substrate Preference	IUCN Status
*Acanthobrama marmid*	Tigris basin	Native	Omnivore	Vegetative	Least Concern
*Alburnoides idignensis*	Tigris basin	Endemic	Omnivore	Vegetative	Not Evaluated
*Alburnus caeruleus*	Tigris basin	Native	Omnivore	Vegetative	Least Concern
*Alburnus doriae*	Namak, Esfahan and Tigris basins	Endemic	Benthivore	Rocky	Not Evaluated
*Alburnus sellal*	Tigris, Kor, Maharlu Lake, Persis and Hormuz basins	Native	Omnivore	Rocky	Least Concern
*Aphanius vladykovi*	Tigris and Esfahan basins	Endemic	Omnivore	Vegetative	Not Evaluated
*Arabibarbus grypus*	Tigris, Persis and Hormuz basins	Native	Omnivore	Vegetative	Vulnerable/Decreasing
*Barbus karunensis*	Tigris basin	Endemic	Omnivore	Rocky	Not Evaluated
*Barbus lacerta*	Tigris basin	Native	Omnivore	Rocky	Least Concern
*Capoeta aculeata*	Tigris and Kor basins	Endemic	Herbivore	Rocky	Not Evaluated
*Capoeta coadi*	Tigris and Esfahan Basins	Endemic	Herbivore	Rocky	Not Evaluated
*Capoeta trutta*	Tigris basin	Native	Herbivore	Rocky	Least Concern
*Carasobarbus kosswigi*	Tigris basin	Native	Omnivore	Rocky	Vulnerable/Decreasing
*Carasobarbus luteus*	Tigris, Persis, Hormuz, Maharlu Lake basins	Native	Herbivore	Rocky	Least Concern
*Carassius gibelio*	Introduced widely; found in all basins of Iran.	Non-native	Omnivore	Vegetative	Not Evaluated
*Chondrostoma regium*	Tigris and Esfahan basin.	Native	Omnivore	Rocky	Least Concern
*Ctenopharyngodon idella*	Introduced widely elsewhere, found in all basins of Iran.	Non-native	Herbivore	Vegetative	Least Concern
*Cyprinion macrostomus*	Tigris basin	Native	Omnivore	Rocky	Least Concern
*Cyprinus carpio*	Native to the Caspian Sea basin. Introduced widely to all basins in Iran.	Non-native	Omnivore	Vegetative	Vulnerable
*Gambusia holbrooki*	Introduced widely elsewhere, found in all basins of Iran.	Non-native	Omnivore	Vegetative	Least Concern
*Garra gymnothorax*	Tigris basin	Endemic	Omnivore	Rocky	Not Evaluated
*Garra rufa*	Tigris, Kor, Maharlu Lake and Persis	Native	Omnivore	Rocky	Least Concern
*Glyptothorax silviae*	Tigris and Persis basins	Endemic	Benthivore	Rocky	Not Evaluated
*Hemiculter leucisculus*	Introduced widely everywhere, found in all Iranian basins.	Non-native	Omnivore	Rocky	Least Concern
*Luciobarbus barbulus*	Tigris and Persis basins	Native	Carnivore	Rocky	Not Evaluated
*Mastacembelus mastacembelus*	Tigris and Persis	Native	Carnivore	Rocky	Least Concern
*Oncorhynchus mykiss*	Introduced widely elsewhere, found in all basins of Iran.	Non-native	Carnivore	Rocky	Not Evaluated
*Oxynoemacheilus freyhofi*	Tigris basin	Endemic	Benthivore	Rocky	Not Evaluated
*Planiliza abu*	Tigris River, Persis, Hormuz and Maharlu Lake basins	Native	Benthivore	Rocky	Least Concern
*Pseudorasbora parva*	Introduced widely everywhere, found in all Iranian basins.	Non-native	Omnivore	Vegetative	Least Concern
*Rhinogobius lindbergi*	Caspian, Namak, Hari and Tigris basins	Non-native	Benthivore	Rocky	Not Evaluated
*Sasanidus kermanshahensis*	Tigris basin	Endemic	Benthivore	Rocky	Endangered
*Squalius berak*	Tigris basin	Native	Omnivore	Rocky	Least Concern
*Squalius lepidus*	Tigris basin	Native	Omnivore	Rocky	Least Concern
*Turcinoemacheilus hafezi*	Tigris basin	Endemic	Benthivore	Rocky	Least Concern
*Turcinoemacheilus saadii*	Tigris basin	Endemic	Benthivore	Rocky	Least Concern
*Capoeta pyragyi*	Tigris basin	Endemic	Herbivore	Rocky	Least Concern

**Table 2.** The composition of fish species in the Karun River basin.

Family/Species	Total Number of Individuals (N)	Total Biomass W(g)	Index of Relative Importance (*IRI*) (%)	Frequency of Occurrence (%)
**Leuciscidae**				
*Acanthobrama marmid*	4	4.78	0.002	1.96
***Chondrostoma regium***	**504**	**10,062**	**10.271**	54.9
***Alburnoides idignensis***	**256**	**2299.47**	**1.229**	17.64
*Alburnus caeruleus*	30	56.93	0.024	3.92
***Squalius berak***	**104**	**5623.12**	**2.771**	39.21
*Squalius lepidus*	76	3630.66	0.371	7.84
*Alburnus doriae*	145	2051.49	0.815	17.64
***Alburnus sellal***	**390**	**4121.19**	**6.786**	60.78
**Cyprinidae**				
***Capoeta aculeate***	**485**	**18,959.85**	**12.416**	47.05
***Capoeta coadi***	**857**	**22,480.05**	**23.004**	62.74
***Capoeta trutta***	**184**	**5104.41**	**2.548**	31.37
*Carasobarbus kosswigi*	15	266.66	0.052	9.80
*Carasobarbus luteus*	42	1280.93	0.077	3.92
*Carassius gibelio*	44	688.52	0.143	
*Cyprinion macrostomus*	148	1612.6	0.839	19.60
*Cyprinus carpio*	9	1348.49	0.055	3.92
***Garra rufa***	**619**	**4532.74**	**10.292**	64.70
***Garra gymnothorax***	**254**	**990.97**	**2.252**	39.21
*Luciobarbus barbulus*	44	825.25	0.528	33.33
***Capoeta pyragyi***	**386**	**16,709.63**	**5.726**	25.49
*Arabibarbus grypus*	2	17.23	0.001	1.96
*Barbus karunensis*	26	786.98	0.213	17.64
***Barbus lacerta***	**79**	**1938.33**	**1.022**	31.37
**Xenocyprinidae**				
*Ctenopharyngodon idella*	1	326.9	0.006	1.96
*Hemiculter leucisculus*	4	21.3	0.004	3.92
**Poeciliidae**				
*Gambusia holbrooki*	9	6.86	0.003	1.96
**Sisoridae**				
*Glyptothorax silviae*	64	296.78	0.613	41.17
**Mastacembelidae**				
*Mastacembelus mastacembelus*	22	1034.19	0.080	5.88
**Salmonidae**				
*Oncorhynchus mykiss*	7	1245.85	0.123	9.88
**Nemacheilidae**				
*Oxynoemacheilus freyhofi*	66	97.85	0.159	11.76
*Sasanidus kermanshahensis*	36	27.81	0.112	15.68
*Turcinoemacheilus hafezi*	173	48.88	0.853	25.49
*Turcinoemacheilus saadii*	36	17.36	0.124	17.64
**Mugilidae**				
*Planiliza abu*	66	1513.9	0.103	3.92
**Gobionidae**				
*Pseudorasbora parva*	3	6.2	0.001	1.96
**Gobiidae**				
*Rhinogobius lindbergi*	5	2	0.002	1.96
**Aphanidae**				
*Aphanius vladykovi*	10	11.72	0.007	3.92

Note: Bold rows show dominant and common species in the Karun River basin.

**Table 3.** Spatial variation in fish species richness, abundance and diversity indices in the Karun River basin.

Site_Code	Shannon−Wiener Diversity Index	Simpson’s Index of Diversity	Margalef Species Richness Index	Pielou Evenness Index	Total Number of Species	Total Abundance
1	1.09	0.58	1.20	0.56	7	151
2	1.58	0.75	1.63	0.81	7	40
3	1.57	0.67	2.28	0.63	12	124
4	1.87	0.82	1.68	0.85	9	118
5	1.72	0.78	1.57	0.88	7	46
6	1.59	0.77	1.27	0.81	7	111
7	1.63	0.79	1.24	0.84	7	125
8	0.76	0.51	0.50	0.69	3	55
9	1.50	0.76	0.92	0.93	6	76
10	1.30	0.59	1.50	0.59	9	205
11	1.32	0.66	1.39	0.68	7	75
12	1.26	0.62	1.77	0.55	10	162
13	0.69	0.61	0.87	0.63	3	10
14	1.29	0.7	0.84	0.93	5	50
16	1.89	0.83	2.15	0.91	8	26
17	0.97	0.51	1.04	0.70	4	18
18	0.69	0.5	1.44	1.00	2	2
19	1.92	0.83	2.08	0.83	10	76
20	2.08	0.83	2.49	0.81	13	123
21	1.73	0.74	1.89	0.83	9	69
22	2.14	0.85	2.38	0.79	15	359
23	1.47	0.74	1.35	0.75	7	85
24	1.94	0.83	2.08	0.84	10	75
25	1.54	0.71	1.29	0.74	8	229
26	1.55	0.7	1.35	0.75	8	177
27	1.01	0.53	1.00	0.63	5	55
28	1.39	0.6	1.90	0.61	10	66
30	1.44	0.71	1.06	0.80	6	110
31	1.84	0.72	2.96	0.70	11	66
32	1.69	0.78	2.04	0.87	7	19
33	2.01	0.83	2.17	0.84	10	95
34	1.88	0.81	2.05	0.78	10	127
35	1.37	0.7	1.38	0.85	5	18
36	1.88	0.79	2.32	0.78	10	123
37	1.30	0.67	1.50	0.59	9	207
38	1.75	0.78	1.85	0.70	12	388
39	1.79	0.78	1.94	0.78	10	103
40	1.87	0.79	2.36	0.78	11	79
41	2.13	0.86	2.91	0.93	8	20
42	1.75	0.82	2.57	0.98	6	7
43	1.46	0.4	2.02	0.63	11	141
44	0.51	0.25	0.63	0.37	4	116
45	1.74	0.75	1.88	0.73	11	202
46	1.10	0.67	1.82	1.00	3	3
47	1.04	0.43	0.65	0.75	4	101
48	1.77	0.8	1.34	0.85	8	184
49	0.00	0.00	0.00	0.00	1	3
50	0.98	0.58	0.62	0.71	4	123
51	0.85	0.49	1.00	0.53	6	152
52	1.55	0.72	1.56	0.75	8	89
53	1.74	0.78	1.98	0.79	9	57
Mean	**1.47**	**0.68**	**1.60**	**0.75**	**7.69**	**102.76**
Range	**0–2.14**	**0–0.86**	**0–2.96**	**0–1**	**0–15**	**0–388**

**Table 4.** Details of measured physico-chemical and habitat parameters in the Karun River system.

Factor	Mean	Min.	Max.	S.D.
Physico-chemical parameters				
pH	7.87	7.03	8.31	0.32
Electrical Conductivity (μS/cm)	475.75	235	2250	281.98
Dissolved Oxygen (mg/L)	8.46	6.05	10.51	0.89
Water Temperature (°C)	18.89	10.7	28.43	3.95
Turbidity (NTU)	43.69	15.93	148.84	26.46
Habitat parameters				
Stream Width (m)	46.49	5	110	24.67
Stream Depth (m)	48.63	27.4	91.9	10.62
Water Velocity (m/s)	3.5	1.5	5.01	0.78
Altitude (m)	1424.94	67	2012	445.05

Note: Mean, minimum (Min.), maximum (Max.) and standard deviations (S.D.) are given.

**Table 5.** Results of CCA for the occurrence of native and endemic fish species and environmental descriptors in the Karun River basin, Iran.

Environmental Descriptors	Axis 1	Axis 2	F-Ratio	*p*-Value
Electrical Conductivity	−0.6787	0.54754	5.4521	0.005 **
Elevation	0.736671	−0.39558	5.205	0.005 **
Water Temperature	−0.51904	0.67974	4.7298	0.005 **
Turbidity	0.169677	0.81554	3.6076	0.005 **
Dissolved Oxygen	0.65749	0.147	3.5576	0.005 **
Water Velocity	0.353703	−0.57765	2.6851	0.005 **
pH	0.441877	0.14455	2.8554	0.01 **
Width	−0.23955	−0.46967	2.1536	0.01 **
Depth	−0.00233	−0.03782	1.4982	0.2

Note:

** = significant at α = 0.05.

## Appendix A. The fish species presence/absence status in the Karun River basin

**Table:**

Family	Cyprinidae															Leuciscidae								Xenocyprididae		Nemacheilidae				Sisoridae	Mugilidae	Cyprinodontidae	Mastacembelidae	Salmonidae	Oxudercidae	Gobionidae	Poeciliidae
Subfamily	Cyprininae							Labeoninae		Barbinae				−		Leuciscinae				Alburninae				Cultrinae	Squaliobarbinae	−				Glyptosterninae	−	Cyprinodontina	−	−	Gobionellinae	Gobioninae	−
Site	*Capoeta coadi*	*Capoeta aculeata*	*Capoeta pyragyi*	*Capoeta trutta*	*Carassius gibelio*	*Arabibarbus grypus*	*Cyprinus carpio*	*Garra rufa*	*Garra gymnothorax*	*Barbus lacerta*	*Barbus karunensis*	*Luciobarbus barbulus*	*Carasobarbus luteus*	*Carasobarbus kosswigi*	*Cyprion macrostomus*	*Chondrostoma regium*	*Squalius berak*	*Squalius lepidus*	*Acanthobrama marmid*	*Alburnus sellal*	*Alburnus caeruleus*	*Alburnus doriae*	*Alburnoides idignesis*	*Hemiculter leucisculus*	*Ctenopharyngodon idella*	*Sasanidus kermanshahensis*	*Turcinoemacheilus saadii*	*Turcinoemacheilus hafezi*	*Oxynoemacheilus freyhofi*	*Glyptothorax silviae*	*PlaniPlaniliza abu*	*Aphanius vladykovi*	*Mastacembelus mastacembelus*	*Oncorhynchus mykiss*	*Rhinogobius lindbergi*	*Pseudorasbora parva*	*Gambusia holbrooki*
S0	−	−	−	−	−	−	−	−	−	−	−	−	−	−	−	−	−	−	−	−	−	−	−	−	−	−	−	−	−	−	−	−	−	−	−	−	−
S1	+	+	−	−	−	−	−	+	+	−	−	+	−	−	−	−	−	−	−	+	−	−	−	−	−	−	−	+	−	−	−	−	−	−	−	−	−
S2	+	+	−	−	−	−	−	+	+	−	−	+	−	−	−	−	−	−	−	+	−	−	−	−	−	−	−	+	−	−	−	−	−	−	−	−	−
S3	+	+	−	+	−	−	−	+	+	−	−	+	−	+	+	+	+	−	−	+	−	−	−	−	−	−	−	+	−	−	−	−	−	−	−	−	−
S4	+	+	−	+	−	−	−	+	+	−	−	+	−	−	+	+	+	−	−	−	−	−	−	−	−	−	−	−	−	−	−	−	−	−	−	−	−
S5	+	+	−	+	−	−	−	+	−	−	−	−	−	−	−	+	+	−	−	+	−	−	−	−	−	−	−	−	−	−	−	−	−	−	−	−	−
S6	+	+	−	−	−	−	−	−	−	+	−	−	−	−	−	+	−	−	−	+	−	+	−	−	−	−	−	−	−	−	−	−	−	+	−	−	−
S7	+	+	−	−	−	−	−	−	−	+	−	−	−	−	−	+	−	−	−	+	−	+	−	−	−	−	−	−	−	−	−	−	−	+	−	−	−
S8	+	−	−	−	−	−	−	−	−	−	−	−	−	−	−	+	−	−	−	−	−	−	−	−	−	−	−	+	−	−	−	−	−	−	−	−	−
S9	+	+	−	−	−	−	−	−	−	+	−	−	−	−	−	+	−	−	−	+	−	+	−	−	−	−	−	−	−	−	−	−	−	−	−	−	−
S10	+	−	−	−	−	−	−	+	+	−	−	−	−	−	−	+	−	−	−	+	−	+	−	−	−	−	+	+	−	+	−	−	−	−	−	−	−
S11	+	+	−	−	−	−	−	+	−	+	−	−	−	−	−	+	−	−	−	−	−	+	−	−	−	−	−	−	−	−	−	+	−	−	−	−	−
S12	+	+	−	−	−	−	−	+	−	−	+	−	−	−	−	+	−	−	−	+	−	+	−	−	−	−	+	+	−	+	−	−	−	−	−	−	−
S13	+	−	−	−	−	−	−	−	−	−	−	−	−	−	−	+	−	−	−	−	−	+	−	−	−	−	−	−	−	−	−	−	−	−	−	−	−
S14	+	+	−	−	−	−	−	−	−	−	−	−	−	−	−	+	−	+	−	−	−	+	−	−	−	−	−	−	−	−	−	+	−	−	−	−	−
S15	−	−	−	−	−	−	−	−	−	−	−	−	−	−	−	−	−	−	−	−	−	−	−	−	−	−	−	−	−	−	−	−	−	−	−	−	−
S16	−	−	+	+	−	−	−	+	+	−	−	+	−	−	−	−	−	−	−	+	−	−	−	−	−	−	−	−	−	+	−	−	−	−	−	−	−
S17	−	−	+	−	−	−	−	+	−	−	−	+	−	−	−	−	−	−	−	−	−	−	−	−	−	−	−	−	−	+	−	−	−	−	−	−	−
S18	−	−	−	+	−	−	−	+	−	−	−	−	−	−	−	−	−	−	−	−	−	−	−	−	−	−	−	−	−	−	−	−	−	−	−	−	−
S19	+	−	−	+	−	−	+	+	+	−	+	−	−	−	−	−	−	−	−	+	−	−	+	−	−	−	+	+	−	−	−	−	−	−	−	−	−
S20	−	−	+	−	−	−	−	+	+	+	+	−	−	−	−	+	−	−	−	+	−	+	+	−	−	+	+	+	−	+	−	−	−	−	−	−	−
S21	−	−	+	−	−	−	−	+	−	+	+	−	−	−	−	+	−	−	−	+	−	−	+	−	−	+	−	−	−	+	−	−	−	−	−	−	−
S22	−	−	+	−	+	−	−	+	+	+	−	−	−	−	+	−	+	−	−	+	−	−	−	−	−	−	−	+	+	+	+	−	−	−	+	+	+
S23	−	−	+	−	−	−	−	+	+	+	−	−	−	−	−	−	−	−	−	+	−	−	+	−	−	−	−	−	−	−	−	−	−	+	−	−	−
S24	−	−	+	−	+	−	−	−	+	+	−	−	−	−	−	−	+	−	−	−	−	−	+	−	+	−	+	+	−	−	−	−	−	−	−	−	−
S25	−	−	+	−	−	−	−	−	−	+	−	−	−	−	−	−	+	−	−	+	−	−	+	−	−	+	−	−	+	+	−	−	−	−	−	−	−
S26	−	−	+	−	−	−	−	−	−	+	−	−	−	−	−	−	+	−	−	+	−	−	+	−	−	+	−	−	+	+	−	−	−	−	−	−	−
S27	−	−	+	−	−	−	−	−	−	+	−	−	−	−	−	−	−	−	−	+	−	−	−	−	−	−	−	−	+	−	−	−	−	−	−	−	−
S28	−	−	+	−	−	−	−	+	−	+	−	+	−	−	−	−	−	−	−	+	−	−	+	−	−	+	−	−	+	+	−	−	−	−	−	−	−
S29	−	−	−	−	−	−	−	−	−	−	−	−	−	−	−	−	−	−	−	−	−	−	−	−	−	−	−	−	−	−	−	−	−	−	−	−	−
S30	−	−	+	−	−	−	−	−	−	+	−	−	−	−	−	−	−	+	−	+	−	−	+	−	−	−	−	−	−	−	−	−	−	−	−	−	−
S31	+	+	+	−	−	−	−	+	+	+	−	+	−	−	−	+	−	−	−	+	−	−	−	−	−	−	−	−	−	+	−	−	−	−	−	−	−
S32	+	+	−	−	−	−	−	+	+	−	−	+	−	−	−	−	+	−	−	−	−	−	−	−	−	−	−	−	−	+	−	−	−	−	−	−	−
S33	+	+	−	−	−	−	−	+	+	−	+	+	−	−	−	+	+	−	−	+	−	−	−	−	−	−	−	+	−	+	−	−	−	−	−	−	−
S34	+	+	−	−	−	−	−	+	−	−	+	+	−	−	−	+	+	−	−	+	−	−	−	−	−	−	−	+	−	+	−	−	−	−	−	−	−
S35	+	+	−	−	−	−	−	−	−	−	+	−	−	−	−	+	−	−	−	−	−	−	−	−	−	−	−	−	−	+	−	−	−	−	−	−	−
S36	+	+	−	+	−	−	−	+	+	−	−	−	−	−	−	+	+	−	−	+	−	−	−	−	−	−	−	+	−	+	−	−	−	−	−	−	−
S37	+	+	−	+	−	−	−	+	−	−	−	−	−	−	+	+	−	−	−	+	−	+	−	−	−	−	−	+	−	−	−	−	−	−	−	−	−
S38	+	+	−	−	−	−	−	+	+	−	+	−	−	−	+	+	+	−	−	+	−	−	−	−	−	−	+	+	−	+	−	−	−	−	−	−	−
S39	+	+	−	+	−	−	−	+	−	−	−	+	−	−	−	+	+	−	−	+	−	−	−	−	−	−	−	−	−	+	−	−	−	−	−	−	−
S40	+	+	−	−	−	−	−	+	−	−	+	+	−	+	−	−	+	−	−	+	−	−	−	−	−	−	+	−	−	−	−	−	−	+	−	−	−
S41	+	−	−	+	−	−	−	+	+	−	−	+	−	+	−	−	+	−	−	+	−	−	−	−	−	−	−	−	−	−	−	−	−	−	−	−	−
S42	−	−	−	+	+	−	−	−	+	−	−	+	−	−	−	−	+	−	−	−	−	−	−	−	−	−	−	−	−	−	−	−	−	−	−	−	−
S43	+	+	−	+	−	−	−	+	+	−	−	+	−	+	+	+	+	−	−	−	−	−	−	−	−	−	+	−	−	−	−	−	−	−	−	−	−
S44	+	−	−	−	−	−	−	+	+	−	−	−	−	−	−	−	−	−	−	−	−	−	−	−	−	−	−	−	−	−	−	−	−	+	−	−	−
S45	+	+	−	+	−	−	−	+	−	+	−	−	−	−	−	+	+	−	−	+	−	−	−	−	−	−	+	−	−	+	−	−	−	−	−	−	−
S46	+	−	−	−	−	−	−	−	−	−	−	−	−	−	−	−	+	−	−	−	−	−	−	−	−	−	−	−	−	+	−	−	−	−	−	−	−
S47	+	−	−	−	−	−	−	−	−	−	−	−	−	−	−	+	+	−	−	+	−	−	−	−	−	−	−	−	−	−	−	−	−	−	−	−	−
S48	+	+	−	+	−	−	−	+	−	−	−	+	−	+	−	+	+	−	−	−	−	−	−	−	−	−	−	−	−	−	−	−	−	−	−	−	−
S49	−	−	−	−	−	−	−	−	−	−	−	−	−	−	−	−	−	−	−	−	−	−	−	+	−	−	−	−	−	−	−	−	−	−	−	−	−
S50	−	−	−	−	−	−	−	+	−	−	−	−	−	−	+	+	−	−	−	−	−	−	−	−	−	−	−	−	−	−	−	−	+	−	−	−	−
S51	−	−	−	+	−	+	−	−	+	−	−	−	−	−	+	−	−	−	−	−	−	−	−	−	−	+	−	−	−	−	−	−	+	−	−	−	−
S52	−	−	−	+	+	−	−	+	−	−	−	−	+	−	+	−	−	−	−	−	+	−	−	−	−	−	−	−	−	+	−	−	+	−	−	−	−
S53	−	−	−	−	+	−	+	−	−	−	−	−	+	−	+	+	−	−	+	−	+	−	−	+	−	−	−	−	−	−	+	−	−	−	−	−	−
